# Did AI get more negative recently?

**DOI:** 10.1098/rsos.221159

**Published:** 2023-03-08

**Authors:** Dominik Beese, Begüm Altunbaş, Görkem Güzeler, Steffen Eger

**Affiliations:** ^1^ Technische Universität Darmstadt, Darmstadt, Hessen, Germany; ^2^ Technische Universität München, München, Bayern, Germany; ^3^ Natural Language Learning Group (NLLG), Faculty of Technology, Bielefeld University, Bielefeld, Nordrhein-Westfalen, Germany

**Keywords:** science-of-science, natural language processing, trend prediction, SciBERT, negativity, citation sentiment

## Abstract

In this paper, we classify scientific articles in the domain of natural language processing (NLP) and machine learning (ML), as core subfields of artificial intelligence (AI), into whether (i) they extend the current state-of-the-art by the introduction of novel techniques which beat existing models or whether (ii) they mainly criticize the existing state-of-the-art, i.e. that it is deficient with respect to some property (e.g. wrong evaluation, wrong datasets, misleading task specification). We refer to contributions under (i) as having a ‘positive stance’ and contributions under (ii) as having a ‘negative stance’ (to related work). We annotate over 1.5 k papers from NLP and ML to train a SciBERT-based model to automatically predict the stance of a paper based on its title and abstract. We then analyse large-scale trends on over 41 k papers from the last approximately 35 years in NLP and ML, finding that papers have become substantially more positive over time, but negative papers also got more negative and we observe considerably more negative papers in recent years. Negative papers are also more influential in terms of citations they receive.

## 1. Introduction

Deep learning has revolutionized machine learning (ML) and natural language processing (NLP) in the last decade. In particular, deep learning has led to unprecedented performance gains on a large number of NLP and ML tasks, including machine translation [1], image classification [2], natural language understanding [3] and text generation [4].

On the other hand, there has seemingly also been a recent surge of papers highlighting limitations of (deep learning) approaches, including claims about models exploiting dataset biases [5], flawed evaluation [6] and general ‘troubling trends’ in ML practice [7].

Indeed, from a historic perspective, deep learning – formerly known under the name of ‘artificial neural networks’ – is a prime exemplar of a technology that has received very mixed assessments over time, ranging from initial hype to negative and positive appraisal in repeating cycles [8,9].

Motivated especially by (recent) observations of negative assessment of individual papers regarding the existing literature and its claims [10], we define a new NLP task of determining the *stance* of a paper (with respect to its related work).^1^ We take a prototypical paper of negative stance to be one that concludes that related work is basically flawed, mistaken and potentially based on false assumptions (cf. table 1); negative papers could also be referred to as *critique papers*, which disrupt current knowledge. By contrast, a prototypical paper of positive stance is one that generally accepts the premises of related work (even though it may identify specific—minor—issues and weaknesses), extends it and sets a new state-of-the-art. Positive papers could also be referred to as *extension papers*, which build upon and extend current knowledge.^2^ (Any particular paper may mix positive and negative elements, so we treat the task as a continuous regression problem.)

**Table 1.** Example of a paper with negative stance. The underlying paper is Niven & Kao [5]. Negative elements are highlighted by us in red.

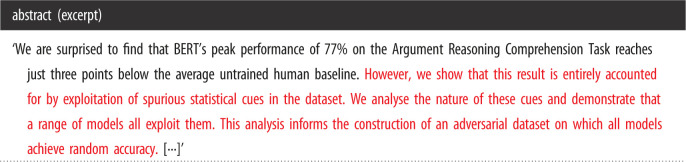

We hold this task important in order to be able to analyse trends in science and its evolution [14], which could potentially anticipate pessimistic future developments (the end of the party). By comparing trends across two core disciplines of artificial intelligence (AI) (arguably one of the most dynamic, promising and intriguing current research fields)—ML and NLP—we can also contrast the evolution and current state of each. The task is also timely, as interest in the analysis of scientific literature in the NLP community has been steadily on the rise recently (cf. §2).

To our best knowledge, we are the first to tackle stance identification on the level of abstracts which, in contrast to individual citations, is (i) more efficient, (ii) less ambiguous, and (iii) focuses on the authors’ core message. We are also the first to measure the evolution of two important scientific subfields (NLP and ML) with respect to stance over time and relate stance to important scientific success measures, i.e. citation counts and acceptance chances at venues. Our contributions:

— we define the new task of stance detection for scientific literature;
— we provide a human-annotated dataset of over 1.5 k scientific papers, labelled for their stance;
— we provide a large-scale trend analysis on over 41 k papers from the NLP and ML community in the past approximately 35 years;
— we address various trend questions including (i) whether negativity has recently increased, (ii) whether positive/negative papers are more influential, and (iii) whether positive/negative papers are more likely to be accepted.

We point out that ‘negativity’, which is a focus of our work, plays a central role in various contexts: in the social sciences, signed social networks are networks in which agents have positive and negative relations to each other, potentially explaining phenomena such as long-term disagreement [16,17]; in science, negative citations may (arguably) be a form of self-correction [18], and publishing negative results may reduce waste on resources for disputed approaches [19]; in economics, the principle of *creative destruction* [20] may explain the progress of capitalism.^3^

## 2. Related work

Historically, analysis of scientific literature is the scope of the fields of *scientometrics* and *science-of-science*. Classical results include the relation between title length and the number of citations a paper receives [21] as well as quantitative laws underlying citation patterns or the number of co-authors of papers over time [22]. Sienkiewicz & Altmann [23] relate textual properties of abstracts and titles of scientific papers to their popularity and find that the complexity of abstracts is positively correlated with citation counts, but abstract and title sentiment (measured as average valence/arousal of all words) are weakly correlated.^4^ In recent years, with the rise in quality of models and approaches, more and more NLP approaches are also devoted to the analysis of scientific literature. We list several relevant studies in the following.

Gao *et al.* [24] ask how much the author response in the ‘rebuttal phase’ of the peer review process influences the final scores of a reviewer, finding its impact to be marginal, especially compared with the scores of other reviewers. Pei & Jurgens [25] study (un)certainty in science communication, finding differences among journals and team size.

Prabhakaran *et al.* [26] predict whether a scientific topic will rise or fall in popularity based on how authors frame the topics in their work. They use a subset of the Web of Science^5^ Core Collection with papers from 1991 to 2010 and analyse abstracts by assigning scientific topics (e.g. stem cell research) as well as rhetorical roles (i.e. scientific background, methods used, results etc.) to phrases. They find that topics that are currently discussed as results and background are at their peak and tend to fall in popularity in the future, whereas topics that are mentioned as methods or conclusions tend to start to rise in popularity.

Arguably the paper most similar to ours is Jurgens *et al.* [27]. They study the entire content of a scientific publication in order to predict its future impact, based on how citations are framed. They distinguish different *functions* of citations: Background (the other work provides relevant information), Uses (usage of data, methods, etc. from the other work), and Comparison or Contrast (express similarities/differences to the other work). They analyse the evolution of the functions over (i) the course of a paper, (ii) different venues, and (iii) time. They show that NLP has seen considerable increase in consensus when authors started to use fewer Comparison or Contrast citations and simply acknowledged previous work as Background. The authors argue that these trends imply that NLP has become a *rapid discovery science* [28], i.e. a particular shift a scientific field can undergo when it reaches a high level of consensus on its research topics, methods and technologies, and then starts to continually improve on each other’s methods. Our approach differs from Jurgens *et al.* [27] in several ways: e.g. we do not analyse individual citations, but directly evaluate the stance of a complete paper (as measured by its framing in the paper’s abstract); most importantly, we are particularly interested in *negative* stances, which as relation is absent in the scheme of Jurgens *et al.* [27].

A number of more recent papers also leverage or study individual citation context, including Cohan *et al.* [29]; Jebari *et al.* [30]; Wright & Augenstein [31]; Lauscher *et al.* [32]. We note that annotating individual citations is more costly than annotating the abstract of a paper, as we do. Annotating individual citations is also a complex task which requires context for disambiguation—as neglected in most previous work [32]—and even then may involve looking up the cited work to understand the citation role. There may also be a bias against direct negative citations of *individual* works, cf. also Bordignon [18]. Sentiment analysis (e.g. polarity) for individual citations is surveyed in Yousif *et al.* [15]. For example, Abu-Jbara *et al.* [13] define a positive citation as one that explicitly states a strength of the target paper. A negative citation points to a weakness and descriptive citations are marked as neutral. We adopt a similar scheme, but apply it to the core message instead of individual citations; we also define positive papers differently^6^—i.e. they make a positive contribution in terms of proposing new techniques extending existing work (thereby implicitly accepting its premises) and setting a new state-of-the-art. Catalini *et al.* [14] study the role of negative citations and find (among others) that they tend to decrease citation counts of the cited paper over time. We study the dual question: whether negative papers (in our sense) receive more citations. Lamers *et al.* [33] study disagreement in science across diverse fields, which is a related concept to that of negative citations, finding that there is highest disagreement in the social sciences and humanities, and lowest disagreement in mathematics and computer science (of which AI is a subfield).

Beyond classification, Yuan *et al.* [34] use NLP models to automatically generate reviews for scientific papers. They conclude that their review generation model is not good enough to fully automate the reviewing process, but could still make the reviewer’s job more effective. Wang *et al.* [35] create automatic reviews for papers by defining multiple knowledge graphs, one extracted from the paper, one from the papers the paper cites, and one for background knowledge. Beltagy *et al.* [36] train a language model (SciBERT; extending BERT) on a large multi-domain corpus of scientific publications.

## 3. Data

We extract our data from two sources: (i) the ACL Anthology^7^ which contains papers and metadata for all major NLP events, and (ii) ML conferences.

### 3.1. NLP dataset

From the ACL Anthology, we extract papers from eight different NLP venues between 1984 and 2021.^8^ For all venues, we only include papers from the main conference and exclude papers from workshops (by manually selecting the volumes) and contributions like book reviews and title indices (by filtering the titles). To extract the data, we download the provided metadata from the ACL Anthology website in the BibTeX format that contains information of authors, title, venue and year. We then use Allen AI’s Science Parse^9^ to extract abstract information and collect citation information from Semantic Scholar.^10^ We refer to this dataset as NLP in the following. NLP contains more than 23 k papers in total. The distribution over the venues is shown in table 2.

**Table 2.** NLP dataset.

venue	no. of papers
ACL	6818
EMNLP	5013
COLING	4647
NAACL	2636
SemEval	2384
CoNLL	1033
CL	952
TACL	351
overall	23 834

### 3.2. ML dataset

We download papers from the respective websites of NeurIPS^11^ , AAAI^12^ , ICML^13^ and ICLR^14^ , and then use Science Parse to extract abstracts and Semantic Scholar to collect citation information as above. ML contains over 18 k papers between 1989 and 2021. The distribution over the venues is given in table 3.

**Table 3.** ML dataset.

venue	no. of papers
NeurIPS	7535
AAAI	4384
ICML	3667
ICLR	2720
overall	18 306

## 4. Data annotation

We annotate the data from NLP and ML for each paper’s **stance (towards related work)**, as given in the authors’ *framing* in a paper’s abstract. In contrast to some related work, we do not annotate stance towards individual citations, but infer the authors’ stance from the paper abstracts and titles as we are interested in the stance of the authors’ *overall core message*. Our focus on title and abstract is a deliberate choice resting on the following observations. (i) In abstracts, authors typically condense the most important information they intend to convey. (ii) This agrees with the insight that abstracts and titles are typically the only piece of the paper that the majority of readers consumes [37]. (iii) Annotating and classifying individual citations is also more costly, cognitively demanding and ambiguous, as outlined in §2. (iv) We are also not interested in whether individual citations are positive or negative but in whether the whole paper (the core message) is framed positively or negatively.

### 4.1. Definition of stance

On a coarse-grained level, we consider three possible stances: (i) we define a prototypical paper of *positive stance* (towards related work) as one that directly or indirectly accepts its premises, builds upon and extends the existing literature and achieves a new state-of-the-art; such a paper contains phrases and sentences such as ‘we present a new approach’; ‘we beat the state-of-the-art’; ‘we release a new dataset’ (cf. appendix table 6). (ii) A prototypical paper of *negative stance* towards related work states that datasets, evaluation protocols or techniques are basically flawed; such a paper contains sentences of negative sentiment such as ‘techniques fail’; ‘approaches are limited’; ‘models are unstable’. (iii) If a paper does not particularly fit into this categorization, e.g. because it discusses or summarizes previous work without criticizing it, then we consider a paper as expressing a *neutral stance*; survey or analysis papers typically fall into this category.

In the annotation, we relax this coarse-grained scheme and instead allow continuous numbers as stances, ranging from −1 (very negative stance) towards +1 (very positive stance), with 0 as neutral stance.^15^ Intuitively, the more negative/positive statements an abstract contains, the more negative/positive it is. The severity of statements also matters for the degree of positivity or negativity, e.g. ‘techniques fail completely’ is more negative than ‘some techniques don’t work properly’. Positive/neutral and negative papers are differentiated by the amount of *direct criticism* they express against existing work: each element of criticism (and its severity) would decrease the positivity of a paper. Neutral and positive papers are differentiated in that the latter has clearly positive elements of advancing the state-of-the-art and providing better solutions.

We provide guidelines for the annotation in the appendix as well as several examples in table 7. In the following, we describe the annotation process and provide statistics.

### 4.2. Annotation statistics and procedure

In total, we manually annotated 1550 papers from NLP and ML. The distribution of papers over venues is given in table 4. In this human-annotated dataset, the ACL conference, which takes place annually since 1979, has most papers (225), followed by NeurIPS (210) and AAAI (205).

**Table 4.** Human-annotated dataset.

venue	no. of papers
ACL	225
NeurIPS	210
AAAI	205
ICLR	196
ICML	167
NAACL	127
EMNLP	123
COLING	111
CL	89
SemEval	50
CoNLL	47
overall	1550

**Figure 1.**
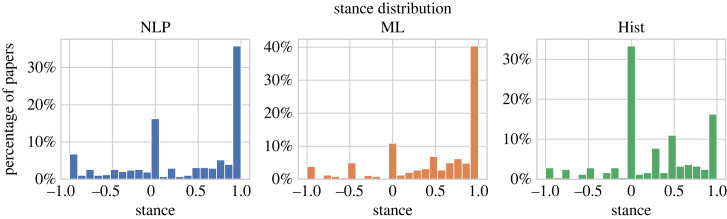
Stance distribution of the human-annotated dataset (Hist: historic papers).

In our human-annotated data, 1018 papers (65.7%) exhibit *positive stance* (greater than or equal to 0.1), 277 papers (17.9%) exhibit *negative stance* (less than or equal to −0.1) and 255 papers (16.5%) are *neutral* (∈(− 0.1, + 0.1)).^16^ We show the more detailed distribution of papers in terms of stance in figure 1.

This statistic does not reflect the true distribution of stance in our data (which is dominated by positive papers, as we will show below), as we oversampled negative papers using heuristics (e.g. looking for particular keywords in abstracts and titles such as *fail* and *limitation* and titles with question marks). Candidates for positive papers were randomly drawn. We did this in order to ensure classifiers trained on datasets that are not too class imbalanced. Manipulating the (training) data distribution is a common approach to ensure better classifiers in the face of small minority classes [38]; our heuristics-based approach also makes annotation much more efficient, as we would otherwise have to annotate considerably more data to obtain negative instances. We note that we *evaluate* our models also on the ‘natural’ data distribution, not only the skewed one; see §6.

### 4.3. Inter-annotator agreement

We had up to four annotators annotate abstracts for stances. The annotators were computer science undergraduate students and one computer science faculty member from NLP. Seventy-one per cent of the human-annotated dataset is annotated by one, 24% by two, 2% by three and 1% by four annotators. This distribution reflects the fact that annotating all instances by all annotators would have been too costly and, given good agreements, also not necessary. Newly incoming annotators first annotated already annotated instances—in order to measure agreement, i.e. their task understanding—then proceeded to annotate independently. We label each abstract’s stance as the average over all the annotators.

**Figure 2.**
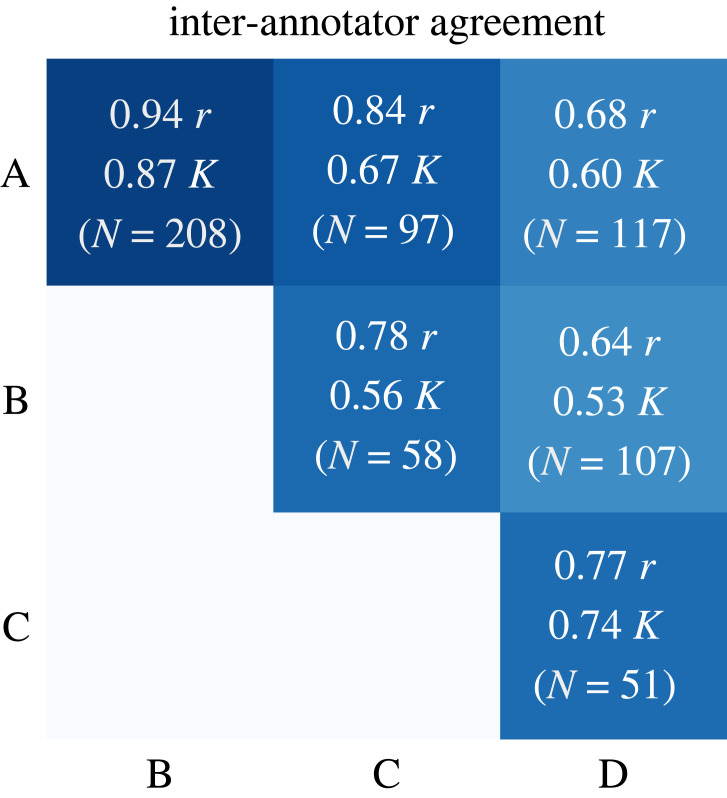
Inter-annotator agreement measured using Pearson’s correlation coefficient (*r*) and Cohen’s kappa coefficient (*κ*), and number of common instances (*N*).

We measure agreement on stance annotation using Pearson’s correlation coefficient, Cohen’s kappa coefficient and Krippendorff’s alpha coefficient. The resulting Pearson correlations among all pairs of annotators (on a common set of instances) range from 0.64 to 0.94 (avg.: 0.77). On a coarse level with three stances, the kappa agreement is between 0.53 and 0.87 (avg.: 0.66). The alpha agreement among all annotators is 0.74 on a ratio scale. Overall, we thus have good agreement. We illustrate inter-annotator agreement (kappa, Pearson) and the number of common instances in figure 2.

### 4.4. Historic versus modern data

We refer to NLP and ML papers published before the year 2000 as *historic* papers (Hist) and papers published since 2000 as *modern* papers. The historic dataset consists of 246 papers, of which 194 belong to NLP and 52 to ML. Modern NLP consists of 578 and modern ML of 726 papers.

## 5. Model

For all our experiments, we use SciBERT [36]. We feed each paper as concatenation of title and abstract separated by special tokens to SciBERT: [CLS] <title> [SEP] <abstract>. We set the maximum token length to 300, which is sufficient for most papers and a good compromise for efficient memory usage. We add a fully connected layer with one output neuron and linear activation on top of the pooled output to obtain a single prediction for the stance of a paper. Since we define stance as a value between −1 and +1, we clip the prediction to the desired range.

The model is fine-tuned with the following hyperparameters: a batch size of 8 or 16, a *slanted triangular learning rate* [39] with a maximum learning rate of 1 × 10^−5^, 2 × 10^−5^ or 5 × 10^−5^, a warm-up ratio of 0.06, and linear decay. We train for 2, 3 or 4 epochs and optimize using Adam [40] with an ε of 1 × 10^−6^, *β*_1_ of 0.9, *β*_2_ of 0.999 and the mean squared error (MSE) as the loss function.

For our experiments in §6, we train 18 models by performing a full grid search over the specified hyperparameters and keep the best model based on the MSE on the dev set. We repeat this five times and calculate performance metrics (cf. §6.1) as the average score of those models.

## 6. Experiments

In the following, we first verify the reliability of our stance detection model described in §5. To do so, we assess its *cross-domain* and *in-domain performance* and compare it with several baselines. Once the quality of the model is assured, we apply it large-scale to determine trends over time and venues in §7.

### 6.1. Experimental set-up

#### 6.1.1. Metrics

We use various metrics to evaluate our models. The **coefficient of determination (***R***^**2**^)** is similar to the MSE but also takes the distribution of the data into account, which makes it more informative and truthful than the MSE [41]. A model that always predicts the expected value has an *R*^2^ score of 0. The range of the metric is (−∞, 1]. The **macro F1 score** is a standard metric to assess the quality of multi-class classification which can take class-imbalance into account. We compute the macro F1 score on coarse-grained stance labels (positive, negative, neutral), see above. We also calculate the **F1 score** for the labels individually. The **natural macro F1 score** samples papers according to the natural distribution of the data (i.e. mostly positive), as predicted by our best performing model. To do this, we draw the test sets randomly (from the existing test sets) according to the true distribution^17^ and then calculate the macro F1 score on the three labels. Since randomness is involved, we repeat this 1 k times and average the scores.

#### 6.1.2. Baselines

We compare our models with simple baselines. **POS**: always predict a positive stance +1; **ZERO**: always predict a neutral stance 0; **NEG**: always predict a negative stance −1; **AVG**: always predict the average of manual annotations.

#### 6.1.3. Cross-domain experiments

Due to the exponential growth of science, our model is mostly trained on more recent data. However, we also want to make sure that we obtain reliable predictions, e.g. for past papers. As a consequence, we first evaluate our model in a cross-domain setting. In this, we train our model on papers from different time stamps or domains and evaluate on a respective out-of-domain test set. For the **source data**, we set the train–dev split ratio to 0.7 and 0.3, and we use the whole annotated data for the **target data**.

#### 6.1.4. In-domain experiments

We also perform in-domain tests where we train and test on the same domain of data (e.g. modern NLP). We set the train–dev–test ratio to 0.6/0.1/0.3.

#### 6.1.5. Combined experiments

We create combined train and dev sets which consist of NLP, ML and Hist papers and evaluate on each domain individually. We set the train–dev–test ratio to 0.6/0.1/0.3.

#### 6.1.6. Data

As mentioned in §4.2, we divided our human-annotated dataset into three groups. The human-annotated NLP dataset consists of a total of 578 papers (60% positive, 16% neutral, 24% negative). The ML dataset consists of 726 annotated abstracts (75% positive, 11% neutral, 14% negative). The NLP portion of the Hist dataset has 194 annotated samples (58% positive, 30% neutral, 12% negative); the ML portion of the Hist dataset contains 52 papers (31% positive, 46% neutral, 23% negative).

### 6.2. Results

**Figure 3.**
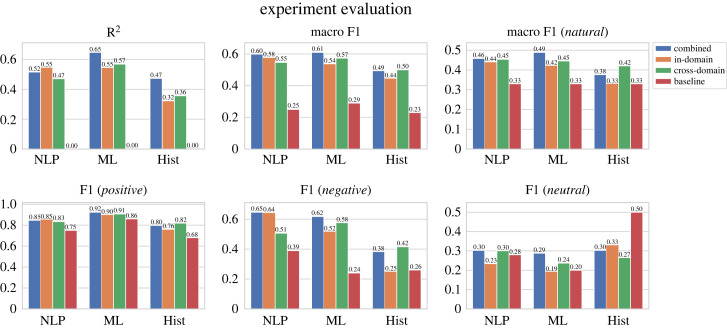
Different metric scores for combined, in-domain and cross-domain evaluation. The *x*-axis represents the test data, and the coloured bars represent different train sets. Cross-domain is the train set which consists of the two remaining datasets. The individual F1 scores in the bottom half refer to the macro F1 score in the top middle. We choose the best baselines for comparison; AVG for *R*^2^, macro F1, macro F1 (*natural*) and F1 (*positive*); ZERO for F1 (*neutral*); and NEG for F1 (*negative*).

Results are shown in figure 3. We observe clear trends: in-domain and cross-domain performance are typically close, but cross-domain performance is better on average (note that cross-domain uses between 1.3 and 6.2 times as much training data as in-domain; cf. table 5). The model trained on combined data, which uses even more data, outperforms in-domain and cross-domain results. In-domain and cross-domain performance on Hist is lowest, which is not surprising as this dataset is smallest in size and assumedly has largest divergence to the modern datasets, due to temporal divergence [42]. On average, the models are best in predicting the positive class, and worst in predicting the neutral class. It is interesting to note that a model trained on a combination of all sources and time periods performs best. It even leads to good results on the Hist portion of our data, which is why we use it for the analysis below.

**Table 5.** Sizes of the train, dev and test sets of the experiments in §6, whose results are shown in figure 3, for combined, in-domain and cross-domain evaluation and NLP, ML and Hist test data.

	combined			in-domain			cross-domain		
	train	dev	test	train	dev	test	train	dev	test
NLP	931	156	173	347	58	173	680	292	578
ML	931	156	217	436	73	217	577	247	726
Hist	931	156	73	148	25	73	913	391	246

### 6.3. Error analysis

**Figure 4.**
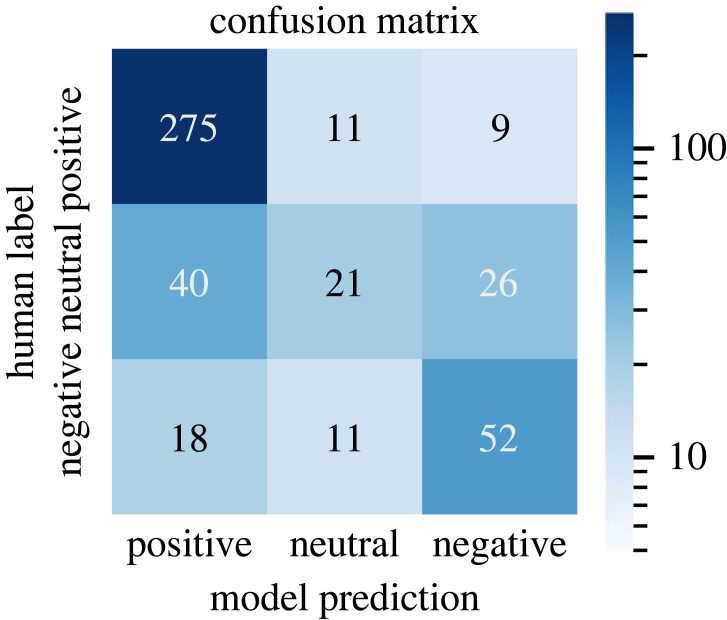
Comparison of human-annotated and model-predicted stance of our best model from the experiments in §6.

We further assess the quality of our best performing combined model by comparing the human annotated and predicted stance in the test set using the confusion matrix shown in figure 4. Positive papers are correctly predicted in most cases. Neutral and negative papers are more frequently confused as positive and when the model predicts a paper to be negative the true class is sometimes neutral.

In appendix table 8, we illustrate sample predictions of our model, randomly sampled from a predicted stance of more than 0.8 (very positive papers) or below −0.7 (very negative papers). We note that the predicted values look very plausible overall. However, especially for the positive papers, the model misses some negative elements, thus overestimates their positivity; arguably the negative papers are also slightly more negative than the model predictions.

We further conduct a manual analysis on papers that have a large discrepancy between predicted and human stance. In these cases, we find that the model often predicts very negative papers as very positive. This may be related to positive papers being the majority class. In several cases, the model was also seemingly led astray by small positive contributions, especially in last sentences, and by superficial cues indicating positive contributions such as *we propose*. One pattern for the misclassified papers is strong criticism followed by the release of a new dataset indicated in the last sentence. In some of the error cases, the gold standard is also incorrect or too extreme, e.g. too strongly negative.

### 6.4. Relation between stance and sentiment

We compare the stance predictions of our model with the predictions of several sentiment analysis tools to assess how similar our concept of ‘stance’ and the concept of ‘sentiment’ are. We test (i) a RoBERTa [43] model for sentiment analysis, called SiEBERT [44], trained on 15 sentiment datasets from diverse text sources (Tweets, reviews, etc.), (ii) VADER [45], a rule-based model for sentiment analysis of social media text, (iii) TextBlob,^18^ a text processing library, and (iv) a valence/arousal lexicon [46]. We evaluate the similarity of stance and sentiment using Pearson’s correlation coefficient, which ranges from 0.01 to 0.27. We interpret the low correlation in two ways: (i) it may be the result of different domains for the training data, which for many sentiment models are texts from social media, while we use scientific texts; (ii) it indicates that our definition of ‘stance’ involves different nuances than simple ‘sentiment’. For example, text such as ‘we propose a new model’ indicates positivity in our context, but may be considered neutral sentiment. Similarly, ‘we identify limitations in existing works’ may be considered neutral sentiment polarity, but indicates negativity in our context.

## 7. Analysis

We analyse large-scale trends from the combined model’s predictions and smooth the graphs with Gaussian blurs.^19^ We further use Welch’s *t*-test [47] and the Kruskal–Wallis *H*-test [48] to detect differences in the distributions and to test our hypotheses; we report the achieved significance levels in parentheses.

Our first questions connect to the recent paper of Bowman [10], who observes ‘a wave of surprising negative results’ in recent years in the NLP community, partly confirming his evidence from selected case studies with our large-scale predictions (but partly also putting it in perspective).

### 7.1. ‘Are there more positive or more negative papers?’

**Figure 5.**
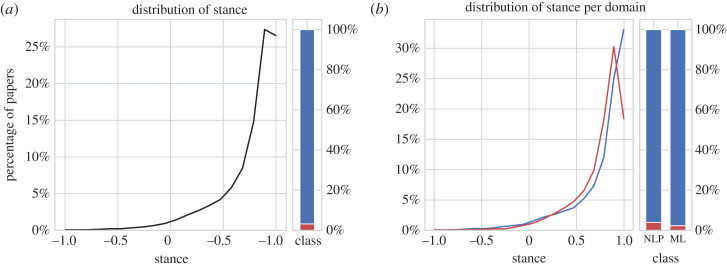
Distribution of stance values. The line plot on the left shows how the stance values are distributed; the bar plot on the right shows the percentage of papers with positive and negative stance.

The histogram of stance values predicted by the model, aggregated over all venues and years, is visualized in figure 5*a*. It shows that most papers have a positive stance and that the more negative the stance gets, the fewer papers there are. Less than 4% of all papers have a negative stance and more than 3/4 of all papers have a stance of greater than or equal to 0.6.

### 7.2. ‘Are NLP papers more positive/negative than ML papers?’

Both hypothesis tests, the *t*-test and the *H*-test, show with a significance level of 0.01% that the distribution of predicted stance values differs between NLP and ML. Figure 5*b* shows the histogram of the predicted stance values, aggregated over all years, for both datasets. The distribution is similar to the overall trend, but ML has more papers with stance values between 0.5 and 0.8, whereas NLP has more papers with a stance of 1.0. Overall, 3.9% of all NLP papers and 2.3% of all ML papers exhibit a negative stance, which makes NLP more negative than ML.

### 7.3. ‘Did AI get more positive/negative recently?’

**Figure 6.**
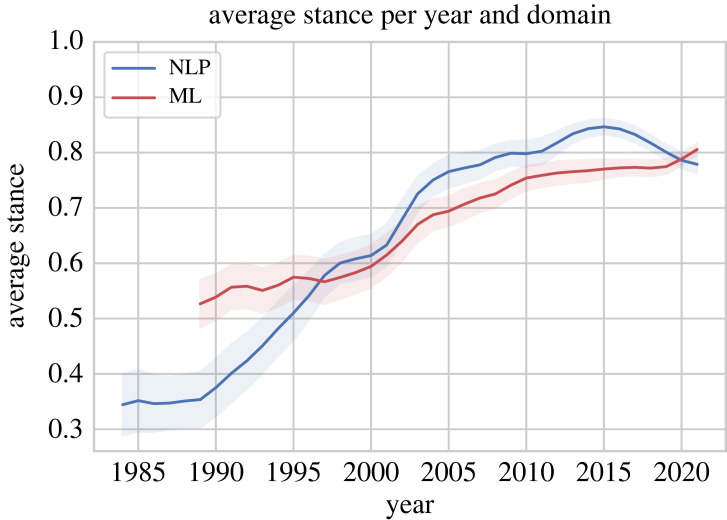
Development of the average stance value over the years for both domains, average stance and 95% confidence interval.

We analyse the development of the *average stance value* over time in figure 6. This shows that the average stance is always positive with a minimum value of 0.34 for NLP in the 1980s. When our ML dataset started in 1989 it was more positive than NLP (*p*-value 0.01%, *t*-test). In the late 1990s, the stance of papers in NLP and ML came closer together, and in the 2000s (when NAACL and SemEval were first held) NLP took over and became slightly more positive than ML (*p*-value 0.01%, *t*-test). The positiveness reached its peak around 2015 with an average stance above 0.80 for NLP. It then started to get more negative for NLP which means that the field of NLP got less positive recently. The ML domain, however, got more positive with a maximum stance of 0.81 in 2021.

Overall, the average stance of both ML and NLP increased substantially from the mid-1980s to the 2020s. This means that papers got more positive on average, i.e. build upon another and report better and better results (i.e. new state-of-the-art performances), confirming the observation that ML and NLP have become ‘rapid discovery sciences’ [27].

**Figure 7.**
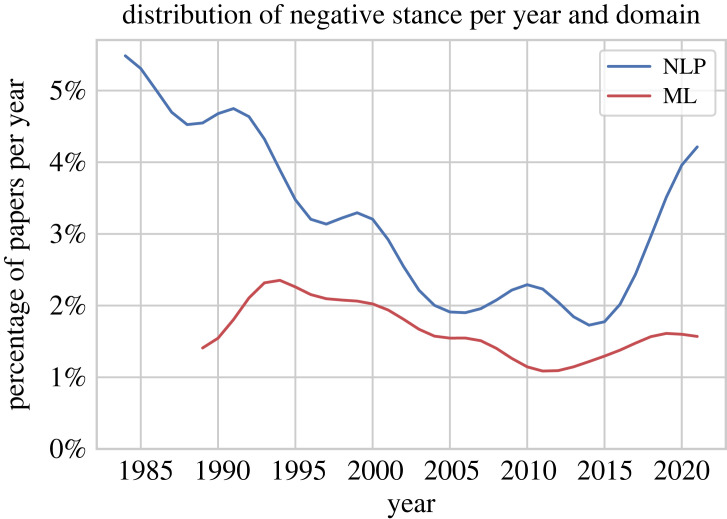
Percentage of papers with a negative stance, calculated as the number of negative papers divided by the total number of papers in each year, for both domains.

We further analyse whether the increase in positiveness from 1990 to 2010 and the decrease in positiveness in the most recent years for NLP comes from more positive/negative papers overall or from positive/negative papers getting less/more positive/negative. Figure 7 shows how many papers have a negative stance in each year. We observe that negativity has peaked in the 1980s and 1990s for NLP and ML, respectively. There was then a continuous downward trend in negativity until the 2010s. In the recent years, negativity has increased for both domains, but considerably more sharply so for NLP, from 2% of all papers in 2015 to 4%.

**Figure 8.**
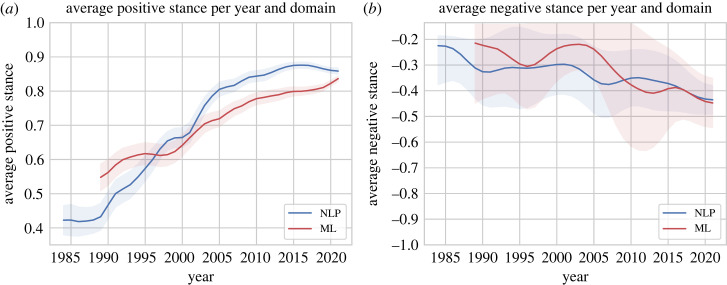
Distribution of the average stance value of papers with a positive (*a*) and a negative (*b*) stance over time for both domains, average stance and 95% confidence interval. The values indicate the average positiveness/negativeness of positive/negative papers.

Figure 8 shows the *average stance value of all positive papers* and the *average stance value of all negative papers* over time. The development of the average positive stance value is very similar to the development of the average stance value overall. By contrast, the average negative stance value has a decreasing trend for both datasets which means that negative papers have become more negative over time.

**Figure 9.**
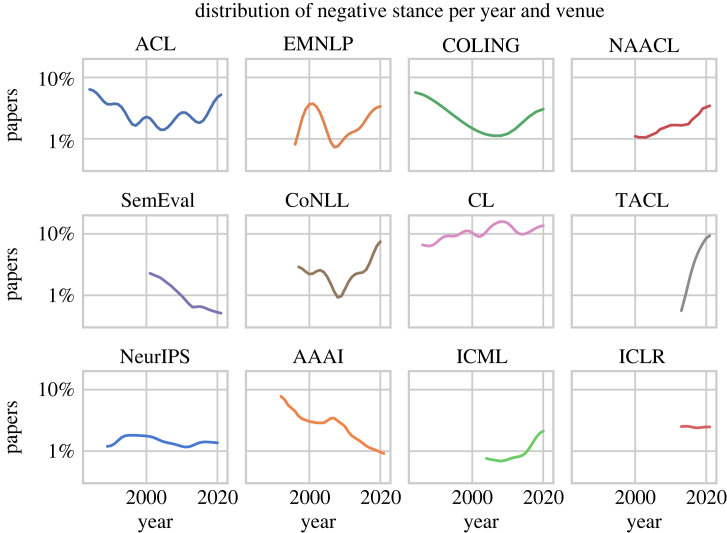
Percentage of negative papers, calculated as the number of negative papers divided by the total number of papers in each year, for each venue on a logarithmic scale.

Finally, we analyse trend curves for individual venues, visualized in figure 9. The trend towards more negative papers in the most recent years is visible for most venues, especially ACL, EMNLP, COLING, NAACL, CoNLL and ICML. TACL has the sharpest increase. SemEval and AAAI do not follow this trend, however. Many venues were more negative before the 2000s and least negative in the 2000s. The CL journal is a noticeable outlier: it is the most negative venue with up to 16% negative papers (*p*-value 0.01%, *t*-test); we note that it is also the only journal in our dataset besides TACL.

### 7.4. ‘Do positive/negative papers receive more/fewer citations?’

**Figure 10.**
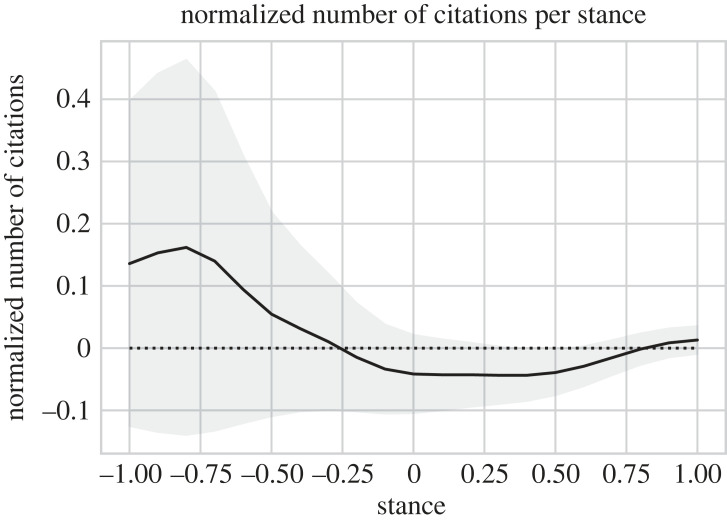
Normalized number of citations a paper with a certain stance value has received, for both domains combined, average number of normalized citations and 95% confidence interval. Normalized values indicate how many citations more or less a paper has received in comparison with the average number of citations a paper published in the same year has received, measured in multiples of the standard deviation of citation counts in that year.

Figure 10 shows how many citations a paper with a certain stance value has received in comparison with papers published in the same year. We compare citation counts using normalized values that indicate how many citations more or less a paper has received in comparison with the average number of citations a paper published in the same year has received, measured in multiples of the standard deviation of citation counts in that year. Positive values indicate more citations than the average, negative values indicate fewer citations. The graph shows that papers with a negative stance of −0.3 or less receive more citations than the average paper in the same year (*p*-value 3%, *t*-test); very negative papers receive even more citations. By contrast, a paper with a positive stance receives less citations on average (*p*-value 6%, *t*-test), but very positive papers with a stance value of more than 0.8 receive slightly more citations (*p*-value 3%, *t*-test).

**Figure 11.**
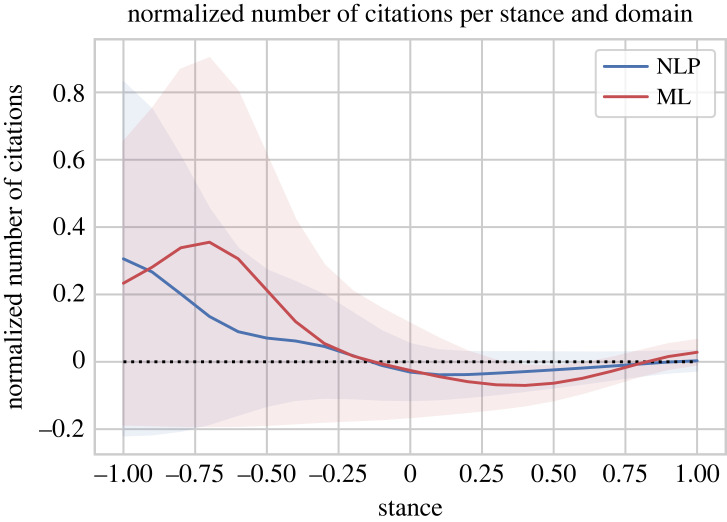
Normalized number of citations a paper with a certain stance and domain has received, average number of normalized citations and 95% confidence interval.

Similar results can be found by analysing the individual domains, NLP and ML, separately, which is shown in figure 11. The domain of ML is more extreme than NLP in that negative papers with a stance of −0.5 or less receive even more citations (*p*-value 6%, *t*-test) and positive papers with stance values from 0.1 to 0.7 even fewer (*p*-value 0.1%, *t*-test).

Overall, this shows that papers of negative stance seem to attract more citations than papers of neutral or positive stance, indicating that they receive more attention and have larger effect on the community. Together with Catalini *et al.* [14], this means that a paper of negative stance receives more citations but decreases the citation counts of papers that it cites negatively, which may indicate that it shifts attention from those papers to itself. The fact that papers of negative stance receive more citations would make stance also suitable for inclusion as feature in models that predict citation count [49].

**Figure 12.**
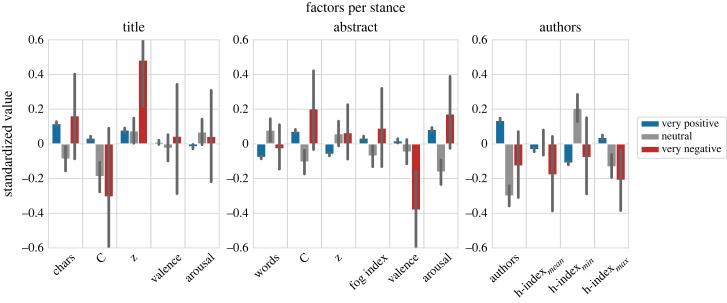
Standardized values for various metrics of very positive, very negative and neutral papers, average value and 95% confidence interval. Metrics are explained in the main text.

However, we acknowledge that also other factors may be at work here, e.g. that stance may only be a confounding variable. For example, it could be that high-prestige authors (e.g. measured in terms of their *h*-indices) tend to be over-represented in papers of negative stance; high-quality authors, in turn, may attract more citations (potentially because they write better papers), which could explain the link between citations and stance. To analyse the relationship between stance and citations in more depth, we performed a linear regression similar to Sienkiewicz & Altmann [23] by measuring various factors (see (a–d) below), including the length and complexity of titles and abstracts, as well as a paper’s stance, to predict its citation count. We could not reproduce their results because the goodness-of-fit of the regression was low, indicating that linear models are not the adequate choice in our case. Instead, we analyse which factors differ between very positive (greater than or equal to 0.8), very negative (less than or equal to −0.8) and neutral papers (∈(− 0.1, + 0.1)) on a subset of 39 k papers for which we have all required metadata, which leads to 24.1 k very positive, 69 very negative and 874 neutral papers. Similarly to Sienkiewicz & Altmann [23], we explore the following factors: (a) **length** (characters in title, and words in abstract), (b) **complexity** (Herdan’s C index [50], *z*-score [51] and Gunning fog index [52]), (c) **sentiment** (average valence/arousal [46]) and (d) **author information** (number of authors, mean/minimum/maximum *h*-index of authors^20^) for papers of positive, negative, and neutral stance. We standardize all factors per venue and show the mean of each factor together with the 95% confidence interval in figure 12. While the length of the title does not reveal a clear trend, longer abstracts are more common in neutral papers. The variance for complexity factors is generally too high to draw conclusions, but titles with a high *z*-score, i.e. high complexity, tend to be more common in very negative papers. Very negative papers have higher arousal and neutral papers have lowest arousal. Author information separates positive, negative and neutral papers best. In contrast to our initial hypothesis expressed above, very negative papers have authors with lower (current) *h*-indices.

### 7.5. ‘Do positive/negative papers have lower/higher acceptance chances?’

**Figure 13.**
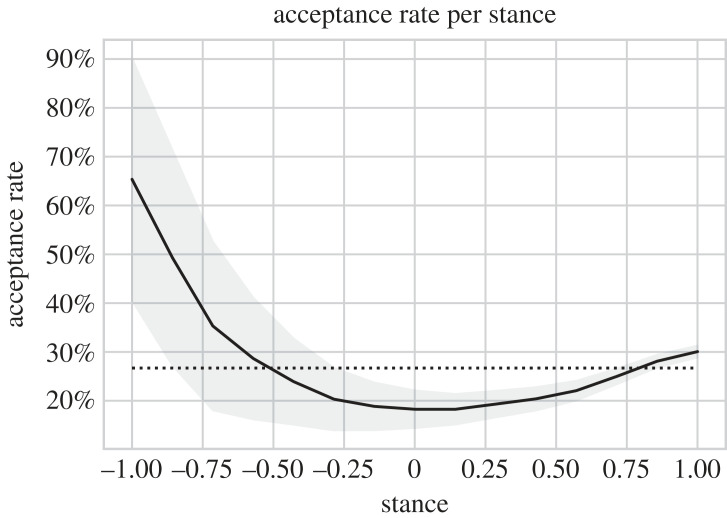
Acceptance rate of a submitted paper with a certain stance value, average acceptance rate and 95% confidence interval. The dotted line indicates the overall acceptance rate of 26.7%.

We use 5453 accepted and 14 974 rejected papers from the ICLR conferences in 2013 and 2017–2021, collected from OpenReview,^21^ and from ACL, EMNLP, NAACL, NeurIPS, AAAI, ICML and ICLR over the years 2007–2017 as collected by Kang *et al.* [53]. The *t*-test and the *H*-test show with a significance level of 0.01% that the distribution of predicted stance values differs between accepted and rejected papers. Figure 13 shows how many papers with a certain stance value were accepted. The trend indicates that papers with a negative stance of −0.6 or less have higher acceptance rates (*p*-value 1%, *t*-test). The acceptance rate for papers with stance values between −0.6 and 0.8 is lower than the overall acceptance rate (*p*-value 0.01%, *t*-test), but for very positive papers, the acceptance rate is slightly higher again (*p*-value 0.01%, *t*-test).

**Figure 14.**
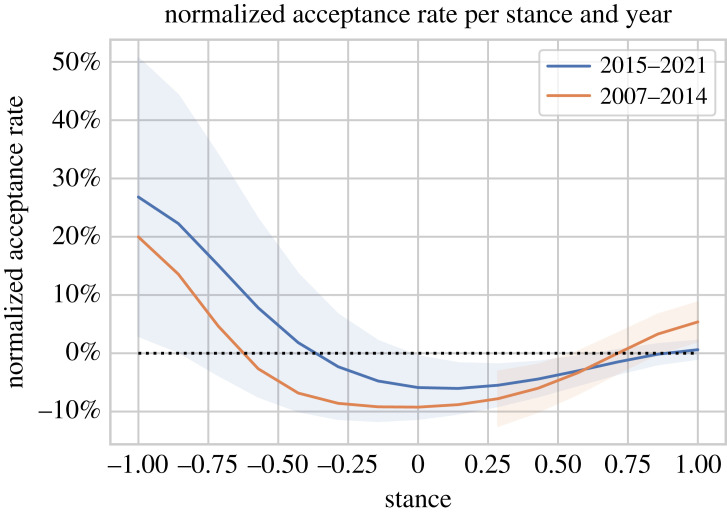
Normalized acceptance rate of a submitted paper with a certain stance value for two time spans, average acceptance rate and 95% confidence interval. Normalized values indicate how many percentage points more or less a paper with a certain stance value is likely to be accepted in comparison with the average acceptance rate in each time span. The dotted line indicates the average acceptance rate.

We also calculate the acceptance rates for two separate time spans, 2007–2014 and 2015–2021, as shown in figure 14. The *t*-test and the *H*-test show that the acceptance chances are different in the two time spans with a significance level of 0.01%. For the most recent years 2015–2021, the trend is similar to the overall trend, including that papers with a very positive stance of more than 0.8 are more likely to be accepted (*p*-value 0.1%, *t*-test), but those papers do not have much higher chances. However, the acceptance rates in earlier years 2007–2014 were different. Papers with a very positive stance of 0.8 or more used to have better acceptance chances than very positive papers in 2015–2021 (*p*-value 0.1%, *t*-test). This is consistent with the trend over time, which shows that fewer papers were negative in the years 2007–2014 than in 2015–2021, implying a bias: positive papers were more popular back then and therefore more positive papers were more likely to be accepted.

## 8. Concluding remarks

We analysed stance in abstracts of scientific publications, where authors position themselves positively or negatively (with respect to related work). We annotated over 1.5 k abstracts from ML and NLP venues and trained a SciBERT model on a subset of the annotated abstracts, verifying that the model is of sufficiently high quality for the task. We then used this model to automatically predict the stance of a paper based on its title and abstract. We applied the model large-scale to a collection of 41 k scientific publications in the domain of NLP and ML from the years 1984 to 2021 to enable large-scale analysis.

The analysis revealed that the majority of papers in the past and today have a positive stance, that the average stance has substantially increased over time, yielding support for the hypothesis that ML and NLP have become ‘rapid discovery sciences’, and that the ML domain is more positive than the NLP domain. Scientific publications used to have a more negative stance in the early days, then became very positive until they started to get more negative again recently. Overall, publications also got more extreme over time, which means that positive papers became more positive and papers with a negative stance more negative. We found (very) negative papers to be more influential than (mildly) positive ones in terms of citations they receive and more likely to be accepted to NLP/ML venues.

We believe that NLP/ML turned more positive when the fields became more statistically solid, starting from the 1990s, and when authors started to build on the existing literature, with a peak of positivity in the mid-2010s, the beginning of the deep learning revolution. This hype has apparently also led to a recent increase in negativity, when papers started to challenge the validity of some of the claims [54], when issues of adversarial robustness [55] and reproducibility [56,57] became apparent and people began to question evaluation frameworks [58].

Our results also inform the recent work of Bowman [10], who warns of the dangers of (what he calls) ‘underclaiming papers’ (which are negative papers in our terminology) by providing quantitative measures of negative papers over time. Given that negative papers tend to receive more attention (in terms of citations), we point out that they may also be key factors in improving the status-quo (cf. also [14]), which highlights their positive contribution to the scientific process. We also note that while negative papers have indeed sharply increased in numbers in the NLP domain (at least) recently, from a historical perspective, they are still on relatively low level.

Future work should address other scientific disciplines beyond NLP and ML for a broader scientific trend analysis, examine the correlation between overall stance of a paper and individual (negative) citations in its related work sections,^22^ annotate word-level rationales for our sentence-level scores, assess the correlation between stance and socio-demographic factors (gender, nationality, affiliation, *h*-index etc.) and analyse how negative papers may potentially transform a field.

We release our data, code and model on GitHub.^23^

## Figures and Tables

**Figure 15.**
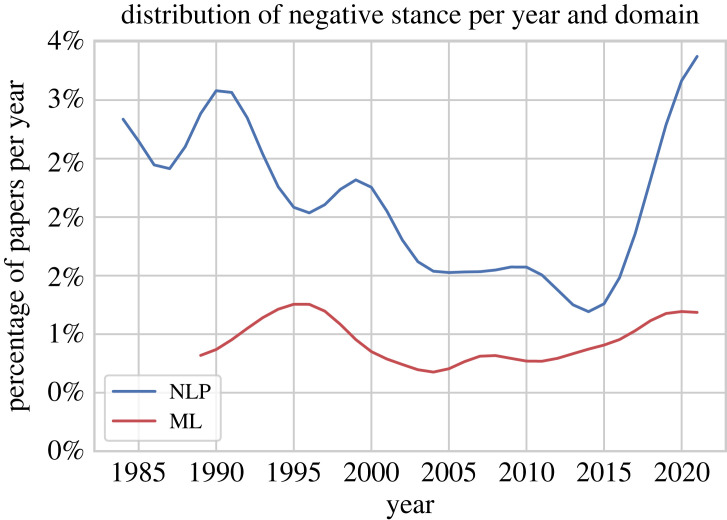
Percentage of papers with a negative stance, calculated as the number of negative papers divided by the total number of papers in each year, for both domains with *alternative thresholds*.

**Figure 16.**
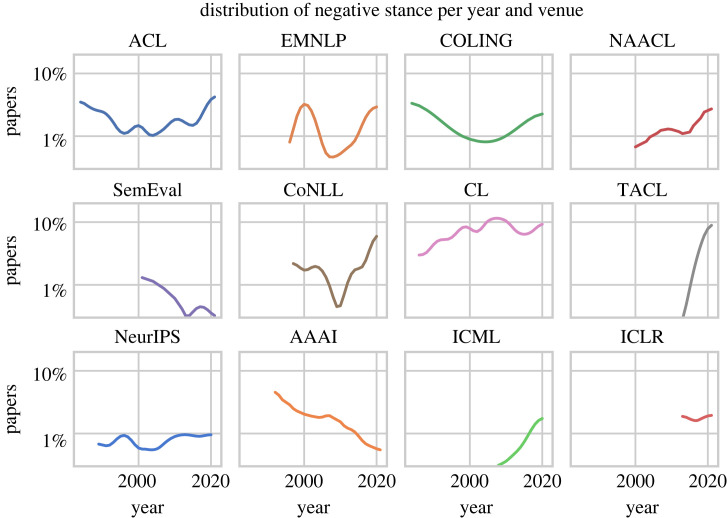
Percentage of negative papers, calculated as the number of negative papers divided by the total number of papers in each year, for each venue on a logarithmic scale with *alternative thresholds*.

## Data Availability

Data and relevant code for this research work are stored in GitHub: https://github.com/DominikBeese/DidAIGetMoreNegativeRecently and have been archived within the Zenodo repository: https://doi.org/10.5281/zenodo.7590214 [74].

## Footnotes

1 This concept is related to positive/negative citations within a paper, which have been annotated in a few works, e.g. Teufel *et al.* [11]; Athar & Teufel [12]; Abu-Jbara *et al.* [13]; Catalini *et al.* [14]; Yousif *et al.* [15]. Our work goes beyond individual citations and assesses the stance of the authors’ main message.

2 We note that there is an asymmetry between positive and negative papers in our definition: while negative papers explicitly relate to related work, positive papers especially relate to themselves, highlighting their own positive contributions. Nonetheless, we take positive papers to implicitly accept the premises of related work, thus they have an *implicit* positive stance to it. We discuss more on this in §4. Calling positive papers incremental, as one reviewer suggested, would seem like a biased judgement concerning their quality.

3 Interestingly, the publication of our paper coincides with a new hype in the AI community surrounding the release of ChatGPT, a very high-quality conversational dialogue model.

4 They use a lexicon lookup to measure valence/arousal, which cannot deal with the contextual usage of words, e.g. the distinction between ‘fail’ and ‘not fail’. By contrast, we learn a model on human annotations to determine the stance of a paper and our notion of stance does also not coincide with sentiment, see §6.4. We finally target very different scientific venues, i.e. ML/NLP versus Web of Science.

5 See https://clarivate.com/webofsciencegroup/solutions/web-of-science/.

6 It would not make sense to define a paper as positive if it *explicitly* lauds previous work as its main message; such papers would probably be rejected as having no additional value to the field of science.

7 See https://aclanthology.org/.

8 We find by introspection that papers before 1984 often have different formatting and may even lack specifically delineated abstracts. For the same reason, we exclude papers from the CL journal before 1986.

9 See https://github.com/allenai/science-parse/. In some instances, that could not be removed automatically, Science Parse included other parts of the paper. This leads to increased uncertainty especially for papers published before the year 2000.

10 See https://www.semanticscholar.org/.

11 See https://papers.neurips.cc/.

12 See https://www.aaai.org/.

13 See https://icml.cc/.

14 See https://iclr.cc/.

15 In practice, annotators typically choose ‘pseudo-discrete’ annotation scores in steps of 0.1, e.g. −0.2, −0.1, 0.0, 0.1, 0.2.

16 In the appendix, we explore how changing the ranges for neutral, positive and negative papers affects our results that relate to a coarse level. In particular, we there define neutral papers as ones that fall into ( − 0.2, + 0.2), with corresponding implications for negative and positive papers. Overall, we find that our results are very similar for such slightly different range definitions.

17 We are aware that the natural distribution, as we define it, relies on our models’ predictions. As these are averages over many instances, we think that our models’ predictions can be relied upon at aggregate level; see our results below.

18 See https://textblob.readthedocs.io/.

19 The observed trends correlate between our model and a model trained as a reference on the *full dataset* with 1.5 k papers (no test split) with a Pearson’s correlation coefficient from 0.76 to 0.99 (avg.: 0.96)

20 We use *h*-indices of a paper’s authors we collected from Semantic Scholar in late 2021, i.e. these are not historic *h*-indices from the time of publication of a paper.

21 See https://openreview.net/.

22 We conducted a small-scale experiment on 20 papers whether a negative paper actually addresses previous work in a negative way by comparing a paper’s stance, predicted using title and abstract, with the stance of the citations, given in the related work or background section of a paper. To do this, we randomly selected 10 very positive and 10 very negative papers based on our model’s predictions and manually annotated the citations (using citation context as in Lauscher *et al.* [32]). In the sample, we find that a paper’s stance and the difference between the amount of positive and negative citations are correlated with a Pearson’s correlation coefficient of 0.62. This means that the stance of a paper may indeed be reflected in the way the authors frame citations in their related work.

23 See https://github.com/DominikBeese/DidAIGetMoreNegativeRecently.

## Appendix A

**A.1. Annotation guidelines**

**Table 6.** Top *n*-grams in the abstract of positive, neutral or negative papers in comparison with abstracts of non-positive, non-neutral or non-negative papers, ranked according to the log-likelihood ratio [59] using the tool from Gao *et al.* [24]. We use the 1.5 k papers from our human-annotated dataset. EOS denotes the end of sentence.

positive *n*-grams	neutral *n*-grams	negative *n*-grams
we propose	computational linguistics	we find
propose a	of the	that these
we propose a	the language	and that
our approach	to be	find that
our method	university of	conclude that
that our	of course	fails to
our model	in the	neural models
a novel	in fact	evidence that
the proposed	the following	we find that
show that our	is not	argue that
paper we propose	is a	our findings
this paper we propose	it is	fail to
we present	of it	adversarial examples
we present a	there is	we show that
experimental results	question of	that the
of our	there are	do not
propose a novel	of some	results suggest that
demonstrate the	the formal	we analyse
we propose a novel	about the	suggest that
we introduce	try to	language understanding
method for	the question of	that this
present a	of natural language	the difficulty
propose an	of a	is not
experiments on	number of	may not
an efficient	the book	to understand
in this paper	there is a	it has been
we propose an	one can	our results
effectiveness of	is no	we conclude that
paper we propose a	the importance of	that there is
method to	the role of	our results suggest
proposed method	properties of the	for this task
that our method	is not the	the ability of
in this paper we	need to be	argue that the
the effectiveness of	it is not	we prove that
demonstrate that our	analysis of the	we investigate the
effectiveness of our	there is no	the case of
algorithm for	a number of	performance EOS EOS
that our approach	the use of	that it is
the effectiveness of our	a large number	a series of
experimental results on	a large number of	the lack of
of the proposed	the fact that	in recent years
paper we present a	part of the	learning EOS EOS
that our model	of the system	we argue that
the proposed method	some of the	with respect to
present a novel	large number of	we show that the

**Table 7.** Selected abstracts from our human annotated dataset. Blue/red phrases denote positive and negative text and are highlighted by us *post hoc*.

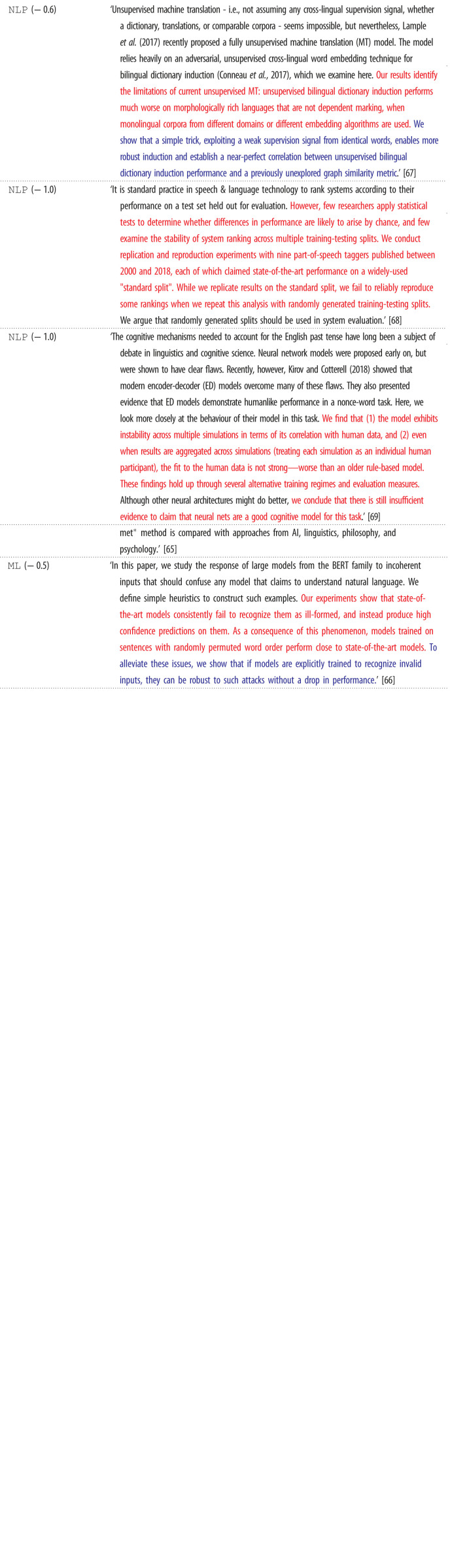

We issued the following guidelines to annotators: (i) the stance is a value in the range from −1 (very negative) to +1 (very positive), and (ii) only the title and abstract of a paper is taken into account.

A contribution has a *positive stance* and is annotated with a positive number up to +1 when: (i) it clearly indicates to improve the state-of-the-art by beating existing standards; (ii) it presents novel techniques; (iii) it proposes solutions to problems of previous work; (iv) it gives insights to existing models or methods and explains why they work.

A contribution has a *negative stance* and is annotated with a negative number up to −1 when: (i) it clearly criticizes previous work for being wrong; (ii) it presents flaws of existing work, i.e. that an approach is deficient with respect to some property; (iii) it analyses errors of other methods and explains why they do not work as expected.

**Table 8.** Predicted highly positive and highly negative papers from our datasets. Blue/red text represents positive/negative rationales; these are added by us.

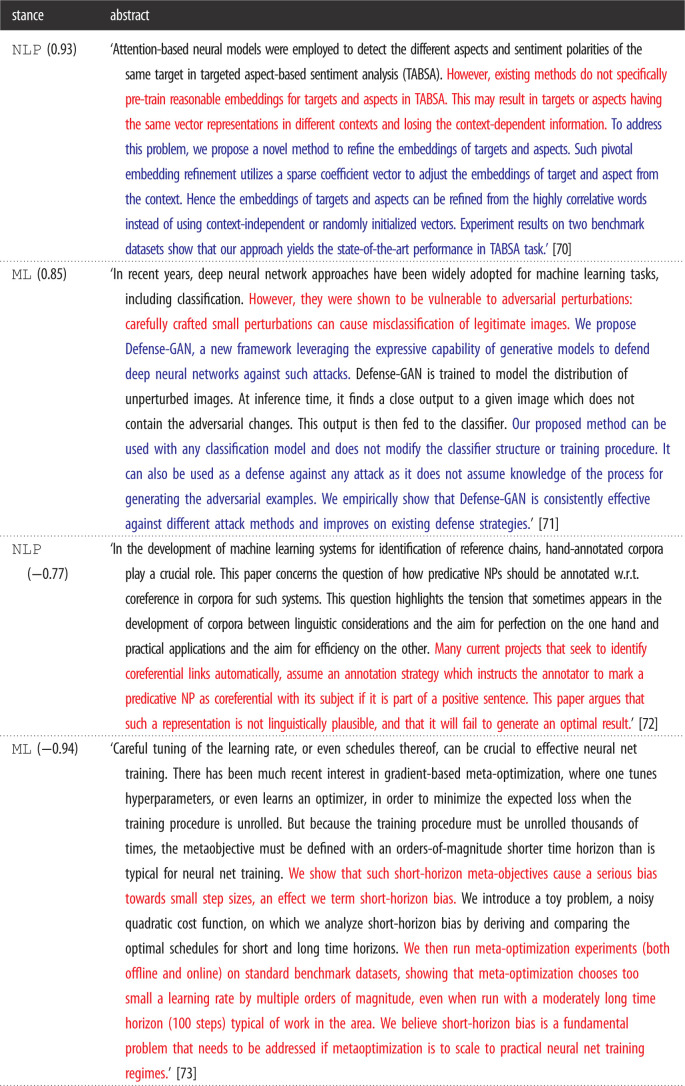

Contributions that have positive and negative parts are annotated with a value between −1 and +1, taking into account the following: (i) the importance of individual parts matters, i.e. ‘some problems’ is less negative than ‘fails to work’; (ii) the amount of positive and negative parts matters; (iii) the last sentence of an abstract is usually the most important sentence. If the last sentence of a contribution is positive, it is more positive than a contribution with a negative last sentence.

Contributions that fall outside this labelling scheme are *neutral* and annotated with a 0. Those include: (i) contributions that explore existing work without beating other systems or explaining why it works or does not work; (ii) contributions that compare, discuss, study or summarize existing work without criticizing it.

**A.2. Range definitions**

We show that our trends are similar with different thresholds for positive (greater than or equal to 0.1), negative (less than or equal to −0.1) and neutral (∈( − 0.1, + 0.1)) papers by recreating figures 7 and 9 with alternative thresholds for positive (greater than or equal to 0.2), negative (less than or equal to −0.2) and neutral (∈( − 0.2, + 0.2)) papers, cf. figures 15 and 16.
